# The OMACS-PIL study: a randomised controlled trial within the OMACS observational study

**DOI:** 10.1186/s13063-019-3958-3

**Published:** 2019-12-27

**Authors:** Lucy Culliford, Rachel Brierley, Madeleine Clout, Rebecca Evans, Rachel Maishman, Dawn Phillips, Hana Tabusa, Barney Reeves, Chris A. Rogers

**Affiliations:** Clinical Trials and Evaluation Unit Bristol, Bristol Trials Centre, Translational Health Sciences, Bristol Medical School, Level 7 Queens Building (Zone A), Bristol Royal Infirmary, Bristol, BS2 8HW UK

**Keywords:** Patient information leaflet, randomised, study within a study, recruitment, consent

## Abstract

**Background:**

There has been little research to investigate whether the appearance of paper patient information leaflets (PILs) used to describe research studies to potential participants influences their decision to take part. Embedding a study within a trial (SWAT) is an efficient way of answering this type of methodological question. We included a randomised SWAT within a large cohort study, Outcome Monitoring after Cardiac Surgery (OMACS), to address this question.

**Methods:**

Potential participants for the OMACS study were randomised to receive one of three PILs, which were identical in content but with varying formatting and use of colour: PIL A (enhanced format), PIL B (hybrid format) and PIL C (standard format). Consent to OMACS was the primary outcome. Consent rates using the three different PIL formats were collected and compared. Qualitative feedback on the different formats was obtained from a public and patient involvement (PPI) group.

**Results:**

For the SWAT, 1517 PILs were sent to potential participants, of whom 640 (42%) consented to take part in OMACS. PIL B had the highest recruitment rate, with 45% of patients consenting to participation; 40% and 41% of patients consented to participation after receiving PILs A and C, respectively. Compared to PIL C, the consent rate was 4% higher with PIL B (45% versus 41%, 95% confidence interval (CI) -2% to + 10%, *p* = 0.16) and 1% lower with PIL A (40% versus 41%, 95% CI − 7% to + 5%, *p* = 0.72).

**Conclusions:**

Consent rates were similar for all three PIL formats. PIL B is being used for the remainder of the host study and will be used to inform the design of PILs for other research studies, as it was the preferred format of the PPI group.

**Trial registration:**

International Clinical Trials Registry, ISRCTN90204321. Registered on 21 January 2015.

## Background

Much research has been conducted on how to improve patient information leaflets (PILs), the consent process, recruitment in randomised controlled trials (RCTs) and response rates to postal questionnaires, which is summarised in several reviews [1–6]. The main focus of that research with respect to PILs has been on the readability or length of PILs [7–15] and use of different media such as audio-visual materials [5] or interactive electronic materials [16], rather than how the appearance of paper PILs can be improved. The outcomes have been patients’ understanding of the study or their satisfaction with the informed consent procedure. There has been less focus on whether recruitment rates can be improved, leading to a lack of evidence in this area. The conclusion of a Cochrane review [4] was that there is currently no clear evidence as to whether modifications to the information presented to participants improves recruitment to RCTs. This conclusion is consistent with that of previous reviews that reported that more research is required [1, 2]. In addition, the design and delivery of information used to invite potential participants into a study has also been identified as a research priority by the Prioritising Recruitment in Randomised Trials study (PRioRiTy) to improve how people are recruited to clinical trials [17].

With respect to the format of the PIL, Reinert and colleagues [11] performed a quantitative and qualitative analysis of study PILs and concluded that information presented to potential participants “needs to be well structured and designed in an appealing manner”. That study focused on how the appearance of a paper PIL might affect the decision to take part in a study, rather than the content. However, efforts were still made to make sure the content was readable and the length of the PIL kept to a minimum. The current research was conducted as a study within a trial (SWAT) by nesting it within a large cohort study, Outcome Monitoring after Cardiac Surgery (OMACS). The objective of the SWAT, OMACS-PIL, was to compare rates of consent using different formats of the host study PIL. Conducting a SWAT is an efficient method of gathering evidence to address uncertainties about the best way to conduct clinical studies and allows methodological evidence to be collected during the main study [18, 19]. In the context of this study, we wanted to make sure that any updates to our PIL were based on evidence. We hypothesised that a PIL with an appealing appearance would be more likely to be read, which would lead to an increased recruitment rate.

## Methods

### The main study

The OMACS study is an ongoing cohort study. Cardiac surgery patients at our institution (University Hospitals Bristol NHS Foundation Trust) are asked to consent to the collection and use of quality of life data, plus data routinely collected about their operation and subsequent recovery. The data collected can then be used in a range of research studies. Patients are approached for consent by post 3 months after their operation. The inclusion criteria for OMACS are adult participants over the age of 18 years who have undergone cardiac surgery at our institution. Around 1000 patients per year are approached to take part. The only patients not eligible to participate are prisoners, patients without the mental capacity to give consent and patients whose main residence is outside the United Kingdom. OMACS is ongoing and the results of the SWAT will be applied to future recruitment to OMACS. This study was chosen as it uses postal consent, so the appearance of the PIL may have more impact than for face-to-face consent, plus the large target population meant that the SWAT could be completed quickly.

### The SWAT

**Fig. 1:**
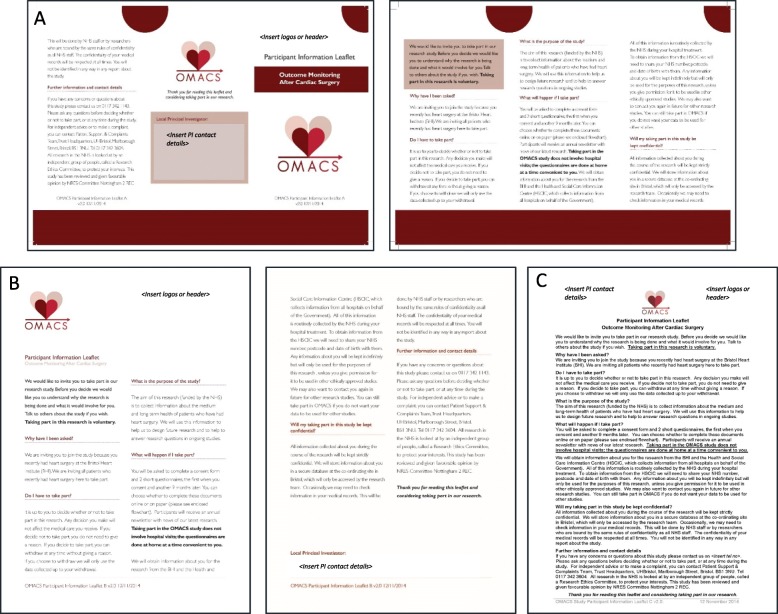
Appearance of the PILs. **a** Tri-fold coloured leaflet produced using a graphic design package, InDesign, by professional printers (enhanced PIL). **b** Coloured A4 sheet produced using a graphic design package, InDesign, by professional printers (hybrid PIL). **c** Black and white A4 sheet produced in Microsoft Word (standard PIL). PIL patient information leaflet

OMACS-PIL was an RCT to investigate whether recruitment to the main OMACS study was affected by the format of the information provided to potential participants. Three different information leaflets were prepared, each with the same content but using different styles and formatting. PIL A (enhanced format) and PIL B (hybrid format) were produced with a specialised graphics package called InDesign (Adobe Systems Incorporated) whereas PIL C (standard format) was produced using Microsoft Word (Microsoft Corporation). PIL A was a full colour tri-fold leaflet, PIL B was a double-sided A4 sheet using columns to break the text up with some coloured headings, and PIL C was a black and white A4 double-sided sheet with standard formatting. Examples of each format can be seen in Fig. 1.

Potential participants were randomly assigned to receive PIL A, B or C in the ratio 1:1:1. The randomisation list was prepared by the study statistician using Stata version 15.1 (StataCorp LP, College Station, TX) and uploaded to the study database. The allocation was linked to the participant record and printed onto the footer of each mail-merged invite letter to allow the correct PIL to be attached. Invitation letters for the main study are prepared for mailing as a monthly batch. To avoid making mistakes in including the correct PIL, the letters were printed in three separate batches, each corresponding to a single PIL allocation. Potential participants were unaware that they were taking part in OMACS-PIL. Members of the study team were not blinded but had no direct contact with potential participants and so were unable to influence consent rates.

### Study population

The study population for OMACS-PIL comprised patients eligible for the OMACS study during the period of the SWAT. With the approval of a National Health Service (NHS) research ethics committee (14/EM/1222), patients were not informed of the SWAT, as knowledge of the SWAT may have influenced how they viewed the PIL. The recruitment period of the SWAT was from 1 June 2016 to 30 September 2017.

### Outcome

The outcome measure was consent to OMACS in each of the groups receiving PIL A, B or C.

### Sample size

Assuming a consent rate of 70% with PIL C (based on historic data from a similar cohort study that ran over several years [20]), then a sample size of 1590 (530 per group) would give 90% power to detect a 10% difference in consent rate between any pair of PIL formats, with an overall significance level of 5% (with a Bonferroni correction for the three comparisons). This was the number of patients to be sent a PIL, rather than the number of patients required to consent. We chose a 10% difference as a compromise between: (a) the large increase in returns of postal questionnaires seen with some measures, such as those with coloured ink (odds ratio 1.39, 95% confidence interval [CI] 1.16–1.67) as reported in the Cochrane review of measures that can increase responses to postal questionnaires [6] and (b) the conclusion of the Cochrane review on increasing participation in clinical trials that there is currently no clear evidence for whether modifications to the information presented to participants improves recruitment to RCTs [4].

### Statistical analysis

The consent rates for the three different PIL formats were described and the differences in consent rate between pairs of PILs were modelled using a logistic regression in Stata version 15.1 (StataCorp LP, College Station, TX). Odds ratios with corresponding 95% CIs are presented for the comparisons between PIL groups with PIL C as the reference category. The analysis took place at the end of the recruitment period for the SWAT.

### Public and patient involvement

To get qualitative information about the PILs, they were presented to the Cardiovascular Public and Patient Involvement (PPI) group of Bristol Biomedical Research Unit, which comprises people who have had cardiac surgery, to elicit their preferences for the format and appearance of the PILs and the reasons for their preferences. The PPI group were not participating in OMACS as they had undergone cardiac surgery before the study started. The group was formed after the start of the SWAT and so did not contribute to the design or content of the PILs, and their observations were obtained whilst the SWAT was taking place. The PPI group had five members and the information was obtained by the PPI coordinator, who was independent of the study. The OMACS chief investigator was present to explain the study.

## Results

For the duration of the SWAT, 1517 invitation letters and PILs were sent to eligible cardiac surgery patients. The numbers of each format of PIL sent were: PIL A 505, PIL B 506 and PIL C 506. The age and sex of the target population are listed in Table 1. The mean age was 64.9 years (standard deviation 13.4), and 1079 / 1517 (71.1%) were male. The average age and proportion of male patients was very similar across the three groups.

**Table 1:** Patient demography by PIL type

PIL	*n* (%)	Mean age in years (standard deviation)	Males, *n* (%)
A	505	64.6 (13.5)	356 (70.5)
B	506	64.7 (14.2)	368 (72.7)
C	506	65.4 (12.5)	355 (70.2)
Overall	1517	64.9 (13.4)	1079 (71.1)

*PIL* patient information leaflet

**Fig. 2:**
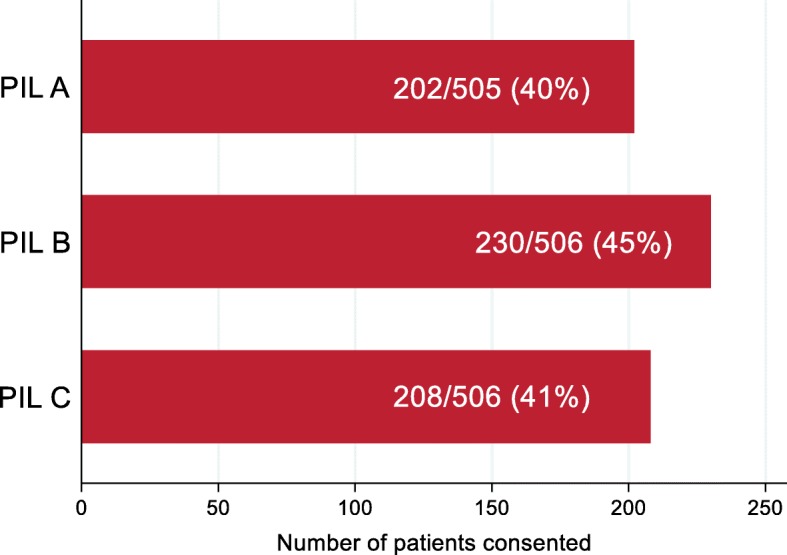
Consent rates by PIL type. PIL patient information leaflet

In total, 640 patients (42%) consented to take part in OMACS. Consent rates across the three PIL formats are shown in Fig. 2. PIL B had the highest recruitment rate, with 45% (95% CI 41–50%) of patients consenting to participation whereas PIL A had the lowest recruitment rate, with 40% (95% CI 35–45%) of patients consenting to participation. The consent rate for PIL C was 41% (95% CI 36–46%). Compared to PIL C (standard format), the consent rate was 4% higher with PIL B (95% CI -2% to + 10%, *p* = 0.16) and 1% lower with PIL A (95% CI -7% to + 5%, *p* = 0.72).

When the different PIL formats were presented to our Cardiovascular PPI group, their general preference was for PIL B, and their least favourite was PIL A. The PPI group felt very strongly that PIL A (enhanced format) looked too professional and looked like advertising material, like leaflets that are posted through letterboxes ‘advertising takeaways’. They also did not like that the study logo took up most of the front page of PIL A, since on receipt of the PIL they would not know what OMACS was. By contrast, for PILs B and C, they could quickly understand that OMACS was a research study. Although the group did not dislike PIL C (standard), on balance they felt that the columnar presentation of PIL B made it more ‘attractive’ and easier to read.

## Discussion

OMACS-PIL has provided some evidence that the formatting of a PIL can influence the perception of a study by potential participants, although the differences in consent rates were not statistically significant. The research team had hypothesised that PIL A would be most appealing to read, as it looked the ‘most professional’, but it was exactly this aspect that the PPI group did not like. The consistency between the quantitative finding and the PPI feedback reinforces the benefit of PPI being an integral part of research.

The sample size achieved was just short of the 1590 target. This is because, due to the lower than expected rates of consent across all PILs, the timing and mode of consent were changed to seeking face-to-face consent at the time of admission for the cardiac operation. The decision to change to face-to-face consent coincided with the sample size for the SWAT almost being met, and the decision was made to halt OMACS-PIL at this point.

The modest increase in recruitment with PIL B is consistent with other research on the design of consent materials. However, much of this previous research has included other interventions alongside changes to the PIL. For instance, in a RCT within the Avon Longitudinal Study of Parents and Children Study, potential participants were randomised to receive one of eight combinations of three interventions: (1) a prior-notification postcard or no contact, (2) a standard or professionally designed consent pack, and (3) a phone or postal reminder [21]. Of these interventions, the most effective was the reminder phone call with a 6.4% higher response rate (95% CI + 2.3 to + 10.6%; *p* = 0.002). The professionally designed consent pack had some impact, increasing response rates by 2.7% (95% CI − 0.06% to 5.5%; *p* = 0.06), but the prior notification postcard had no effect. Phone call reminders can have significant resource implications, hence our decision not to include this as an intervention.

The START programme run by the Medical Research Council (MRC) also looked at the format of paper PILs, but this was an enhanced PIL where the wording was refined after several rounds of user testing [12, 13]. This approach has resource implications and of the work published so far, there have been only marginal improvements in recruitment [12, 15]. The Healthlines studies showed modest increases in a positive response to an invitation to participate in a study using the enhanced PIL, which, like OMACS-PIL, were not statistically significant (19% versus 16%, difference of 2.9%, 95% CI − 1.1% to 6.9% in the Healthlines depression study, *n* = 1364, and 24.0% versus 21.9%, difference of 2.0%, 95% CI − 4.3% to 8.4% in the Healthlines cardiovascular disease study, *n* = 671) [12]. In Early CDT Lung Cancer Scotland, the proportion of patients who positively responded to the invitation was 224 / 1136 (19.7%) in the intervention group (optimised PIL) and 205 / 1126 (18.2%) in the control group (difference of 1.5%, 95% CI  − 1.7% to 4.7%) [15]. In addition to these MRC Start studies, the REFORM study concluded that there was limited evidence of the benefit of using optimised information materials on recruitment and retention rates [14] and the recently updated Cochrane review of methods to enhance recruitment to RCTs concluded that: ‘Using a tailored, user-testing approach to develop participant information leaflets makes little or no difference to recruitment’ [22]. There has been a Cochrane review of methods to increase response rates to postal questionnaires [6], but this focused on the design of the questionnaire or the method of postage (e.g. first-class post) and not on the PIL provided to participants.

Resource implications for developing electronic media and the limited existing evidence that these media can improve recruitment [5] were the reasons that a multi-media approach was not explored in OMACS-PIL.

One area for concern in OMACS is that the overall response rate was much lower than expected. The expected response rate was based on similar work involving postal consent of cardiac surgery patients (Long-Term Monitoring Study), which had been as high as 80% [20]. There are a few differences between the studies: the timing of the approach for consent was at 3 months versus 12 months after cardiac surgery, and the packs sent to OMACS patients had more documents and offered more choices in how to participate. The Long-Term Monitoring Study was conducted purely by post, whereas OMACS allowed participants to register, consent and complete questionnaires online if they preferred. This extra complexity may have contributed to the lower consent rate. The options were offered to allow participants to complete the study in the way most convenient to them, but it may be that the extra thinking involved meant that they did not take part at all. Again, this shows that researchers should not assume they know what patients want.

A limitation of this study is that not all of the three PIL formats would be suitable for all studies. The amount of information that can be contained in the format of PIL A is constrained. This was not a problem for OMACS as the information required to be conveyed about OMACS was limited, since OMACS is an observational study and not an RCT, and therefore was easily incorporated into the different formats. The format of PIL A may not be suitable for interventional trials where more information about the intervention and risk and benefits need to be included. Even for OMACS, the volume of information included in the PIL has increased since the SWAT was conducted, due to the requirements of NHS Digital for hospital episode statistics, which is a feature of the main study. The extra information was easily incorporated into PILs B and C but would have been more challenging to incorporate into PIL A due to the way that the information was presented and formatted. To make PIL B easier to use in OMACS and other studies and to remove the need for specialist software, we have now recreated the format in Microsoft Word (Microsoft Corporation), and this format is now being used for the host study.

The PPI work included was informal and the PPI group was relatively small. However, their opinions were consistent with the quantitative finding of the SWAT. The PPI group were not involved in the design of the PILs. However, the content of the PIL is under review and their views will be considered for future versions. We did not inform the potential participants about the SWAT as we felt that it would influence how they viewed the PIL, so we were unable to elicit potential participant views about the PIL formats.

A strength of OMACS-PIL is that the formatting of the PIL is something that can be improved in any study. A positive improvement in recruitment rates does not require rounds of user acceptability testing, as required by other projects (e.g. MRC START), or extra resources, as required with telephone contact or a multimedia approach, and could be achieved with PPI input. This means that it can be implemented with minimal or no impact on the study budget. OMACS-PIL was randomised and the participants were unaware of the SWAT. However, based on the results of OMACS-PIL and the other similar studies, any improvement in consent rates are likely to be small.

## Conclusions

The hybrid PIL (PIL B) was preferred by the PPI group and resulted in a slightly higher consent rate to the host study than PIL A (enhanced format) or PIL C (standard format), although this difference was not statistically significant. PIL A, which was the most expensive to produce, was the least preferred option for the PPI group and did not improve consent rates. PIL B is now being used as the PIL for the remainder of the OMACS study, and the findings from this study are being used to inform the design of PILs for interventional trials. The work also highlighted the importance of involving PPI groups in the design of information aimed at potential study participants.

## Data Availability

The datasets used or analysed during the current study are available from the corresponding author on reasonable request.

## Abbreviations

CI: Confidence interval
MRC: Medical Research Council in the UK
NHS: National Health Service in the UK
OMACS: Outcome Monitoring after Cardiac Surgery
PIL: Patient information leaflet
PPI: Public and patient involvement
RCT: Randomised controlled trial
SWAT: Study within a trial
